# Green synthesis and crystal structure of 3-(benzo­thia­zol-2-yl)thio­phene

**DOI:** 10.1107/S2056989017014530

**Published:** 2017-10-13

**Authors:** Linh Nguyen Ngoc, Trung Vu Quoc, Hoan Duong Quoc, Manh Vu Quoc, Luong Truong Minh, Chien Thang Pham, Luc Van Meervelt

**Affiliations:** a Faculty of Chemistry, Hanoi National University of Education, 136 Xuan Thuy, Cau Giay, Hanoi, Vietnam; b Faculty of Foundation Science, College of Printing Industry, Phuc Dien, Bac Tu Liem, Hanoi, Vietnam; c VNU University of Science, Department of Inorganic Chemistry, 19 Le Thanh Tong Street, Hoan Kiem District, Hanoi, Vietnam; d Department of Chemistry, KU Leuven, Biomolecular Architecture, Celestijnenlaan 200F, Leuven (Heverlee), B-3001, Belgium

**Keywords:** crystal structure, thio­phene, benzo­thia­zole, microwave-assisted synthesis, solvent-free, whole-mol­ecule disorder

## Abstract

The title compound, C_11_H_7_NS_2_, was prepared in high yield (87%) using a solvent-free microwave-assisted synthesis. The structure shows whole-mol­ecule disorder with occupancies for two orientations (A and B) of 0.4884 (10) and 0.5116 (10), respectively. The thio­phene and benzo­thia­zole rings are almost planar and make dihedral angles of 10.02 (18) and 12.54 (19)° for orientations A and B, respectively. Slipped π–π stacking between the aromatic rings, together with C—H⋯π, C—H⋯S and C—H⋯N inter­actions, result in a herringbone motif in the crystal packing.

## Abstract 2

A solvent-free microwave-assisted synthesis of the title compound is presented together with its crystal structure which is characterized by the herringbone motif in the packing.

## Chemical context   

Thio­phene-containing heterocycles have many applications in pharmacology, such as anti-inflammatory and analgesic agents (Issa *et al.*, 2009 ▸), electrochromic and electronic devices (Elbing *et al.*, 2008 ▸), and polyelectrolytes-based water-soluble sensing agents for the detection of DNA, proteins and small bioanalytes (Ho *et al.*, 2008 ▸; Feng *et al.*, 2008 ▸). Benzo­thia­zole-based compounds have attracted much attention in recent times due to their wide-ranging biological activities, such as anti­cancer, anti­fungal and anti­bacterial activities (Aiello *et al.*, 2008 ▸; Cho *et al.*, 2008 ▸). In addition, some other 2-amino­benzo­thia­zole derivatives showed anti­bacterial, anti-inflammatory and analgesic properties (Bhoi *et al.*, 2014 ▸). A novel poly 3-(benzo­thia­zol-2-yl)thio­phene-based conductive poly­mer has been synthesized by chemical and electrochemical polymerization (Radhakrishnan *et al.*, 2006 ▸; Radhakrishnan & Somanathan, 2006 ▸). These polymers were studied for their photoabsorption and photoluminescence characteristics and were investigated in polymeric light-emitting diodes. Some synthetic methods developed for preparing 3-(benzo­thia­zol-2-yl)thio­phene are available using a mixture of thio­phene-3-carbaldehyde and *o*-amino­thio­phenol refluxed in ethanol (Esashika *et al.*, 2009 ▸) or a mixture of 3-bromo­thio­phene, magnesium turnings and 2-chloro­benzo­thia­zole (Radhakrishnan *et al.*, 2003 ▸). 2-Substituted benzo­thia­zoles have been synthesized through condensation of bis­(2-amino­phen­yl) di­sulfides with aryl­aldehydes catalyzed by NaSH under microwave irradiation (Liu *et al.*, 2017 ▸). X-ray single-crystal structure determinations of two (1,3-benzo­thia­zol-2-yl)thio­phene derivatives synthesized from phenyl iso­thio­cyanate (Fun *et al.*, 2012 ▸) and benzo­thia­zole (Cheng *et al.*, 2016 ▸) have been reported, as well as of 4-(1,3-benzo­thia­zol-2-yl)thio­phene-2-sulfonamide complexed with cyclin-dependent kinase 5 (Malmström *et al.*, 2012 ▸). However, 3-(benzo­thia­zol-2-yl)thio­phene itself has not been studied by crystallographic methods. In this study, we present a solvent-free microwave-assisted synthesis of 3-(benzo­thia­zol-2-yl)thio­phene, starting from thio­phene-3-carbaldehyde and *o*-amino­thio­phenol, together with its crystal structure determination. The reaction was performed in a short time, without solvent and catalyst, leading to a simple purification protocol and a high yield (87%).
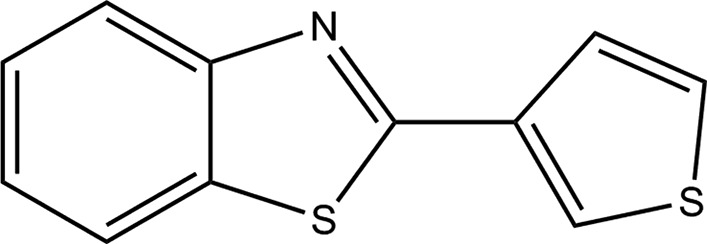


## Structural commentary   

**Figure 1:**
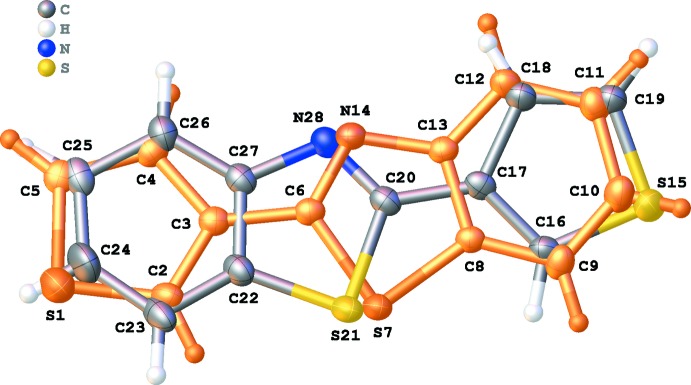
View of the asymmetric unit of the title compound, showing the atom-labelling scheme. Displacement ellipsoids are drawn at the 50% probability level. H atoms are shown as small circles of arbitrary radii. Orientation A of the disordered compound (occupancy factor 0.488) is shown in orange.

The title compound crystallizes in the monoclinic space group *P*2_1_/*c* with four mol­ecules in the unit cell. The structure exhibits whole-mol­ecule disorder by a rotation of approximately 180° around an axis running close to the S and N atoms of the benzo­thia­zole ring, resulting in two orientations (A and B) of about the same shape (Fig. 1 ▸). In addition, orientations A and B both have similar occupancies of 0.4884 (10) and 0.5116 (10), respectively. All the heterocyclic rings are almost planar, with r.m.s. deviations of 0.017 (thio­phene ring S1–C5), 0.004 (thio­phene ring S15–C19), 0.010 (benzo­thia­zole ring C6–N14) and 0.021 Å (benzo­thia­zole ring C20–N28). For orientation A, the angle between the best planes through the thio­phene and benzo­thia­zole rings is 10.02 (18)°. In orientation B, this angle is 12.54 (19)°. The relatively planar structure of the compound results in intra­molecular S⋯H contact distances shorter than the sum of the van der Waals radii of S and H (S7⋯H2 = 2.849 Å and S21⋯H16 = 2.824 Å).

## Supra­molecular features   

**Figure 2:**
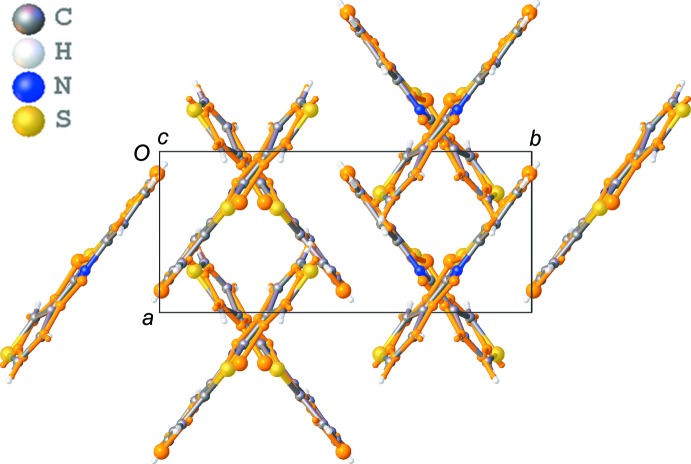
Crystal packing of the title compound shown in projection down the *c* axis. Orientation A of the disordered compound (occupancy factor 0.488) is shown in orange.

**Figure 3:**
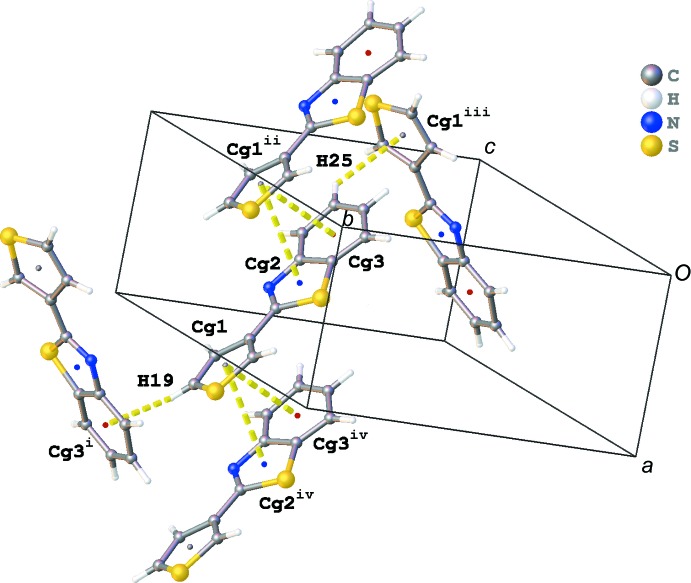
Slipped π–π stacking between the aromatic rings and C—H⋯π inter­actions for orientation B. [Symmetry codes: (i) −*x* + 2, *y* + 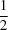, −*z* + 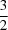; (ii) *x* − 1, *y*, *z*; (iii) −*x* + 1, *y* − 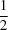, −*z* + 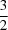; (iv) *x* + 1, *y*, *z*.]

**Figure 4:**
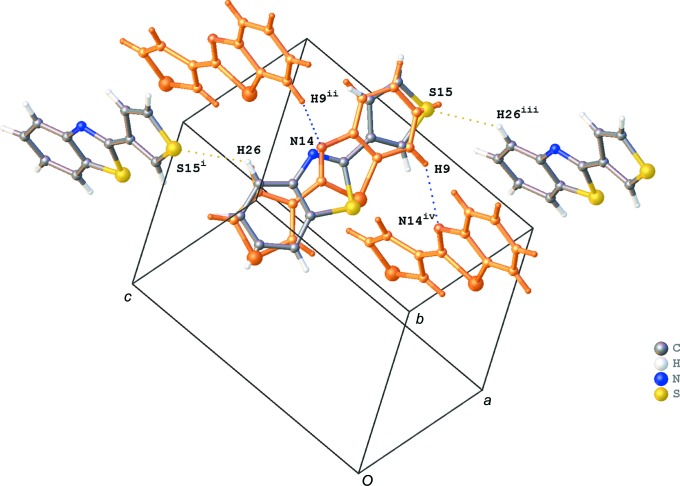
Infinite chain formation through C—H⋯N (blue dashed lines) and C—H⋯S (yellow dashed lines) interactions in the crystal packing of the title compound. Orientation A of the disordered compound (occupancy factor 0.488) is shown in orange. [Symmetry codes: (i) *x* − 1, −*y* + 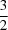, *z* + 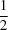; (ii) *x*, −*y* + 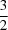, *z* + 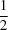; (iii) *x* + 1, −*y* + 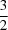, *z* − 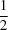; (iv) *x*, −*y* + 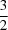, *z* − 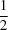.]

The crystal packing of the title compound shows a herringbone motif (Fig. 2 ▸). This motif is built up by slipped π–π stacking between the aromatic rings and C—H⋯π inter­actions. The shortest centroid–centroid distances (*Cg*⋯*Cg*) observed in the π–π stacking for orientation B are shown in Fig. 3 ▸ and are listed in Table 1 ▸ for both orientations. The stacking mol­ecules inter­act further with neighbouring mol­ecules through C—H⋯π inter­actions (Fig. 3 ▸ and Table 2 ▸). In addition, infinite chains running in the [201] direction are formed through C—H⋯N and C—H⋯S inter­actions (Fig. 4 ▸ and Table 2 ▸). The crystal packing contains no voids. Whole-mol­ecule disorder is usually caused by a packing which is determined by van der Waals inter­actions only or by a lack of directional inter­actions in the packing. However, the crystal packing of the title compound shows several directional inter­actions, and hence the whole-mol­ecule disorder is the consequence of the very similar inter­ations with neighbouring mol­ecules for the two orientations.

**Figure 5:**
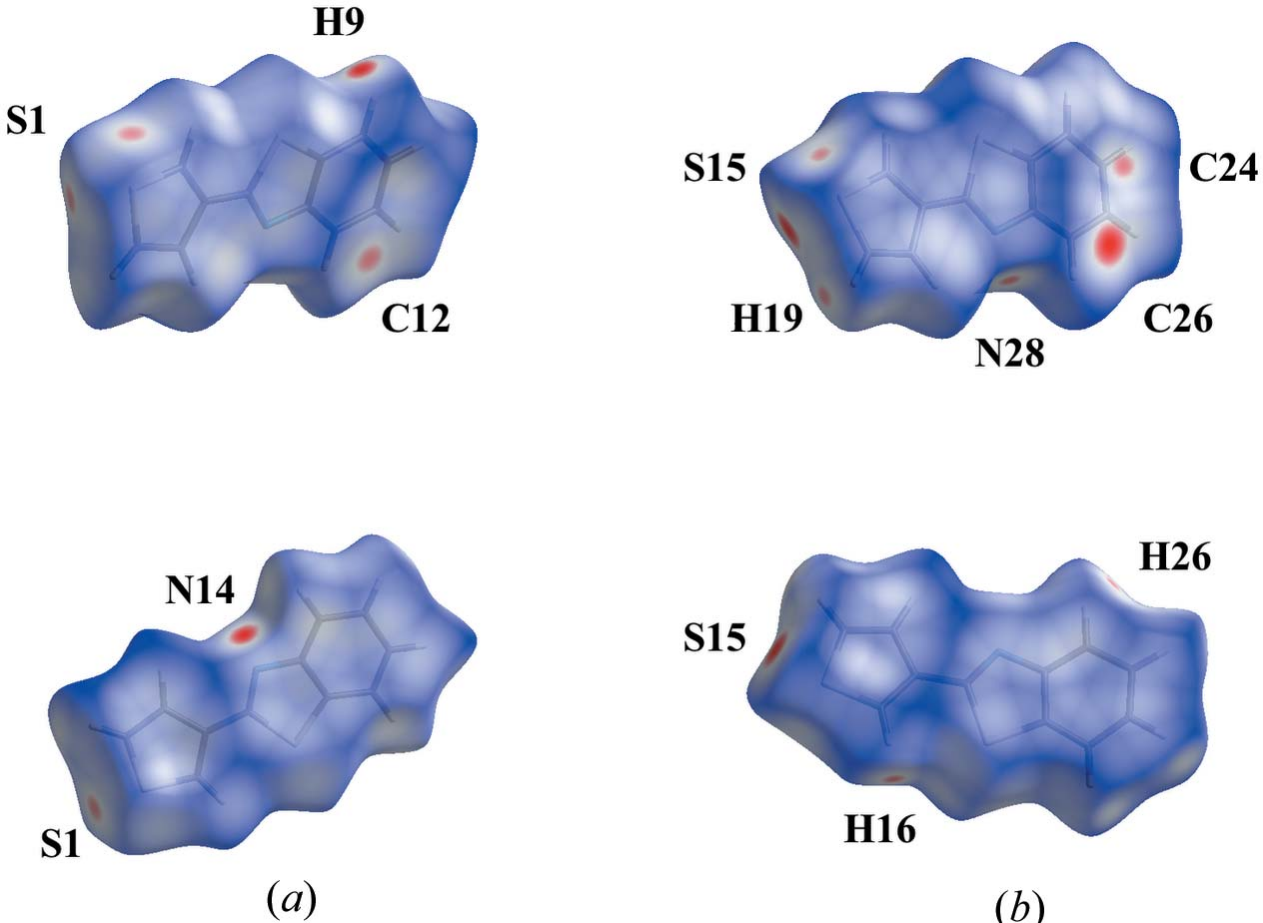
Two views of the Hirshfeld surfaces mapped over *d*_norm_ for (*a*) orientation A in the range −0.151 to 1.099 a.u. and (*b*) orientation B in the range −0.134 to 0.936 a.u.

Additional insight into the inter­molecular inter­actions was obtained from an analysis of the Hirshfield surface and two-dimensional fingerprint plots using *CrystalExplorer* (McKinnon *et al.*, 2007 ▸; Spackman & Jayatilaka, 2009 ▸). Fig. 5 ▸ illustrates the Hirshfeld surfaces mapped over *d*_norm_ for both orientations. The bright-red spots near atoms H9 and N14 for orientation A and near atoms H26 and S15 for orientation B are indicative for the hydrogen bonds given in Table 2 ▸. For orientation A, the red spots near atoms S1 and C12 refer to short C⋯S/S⋯C contacts and in the case of S1 also S⋯S contacts. The red spots for orientation B near atoms N28 and H16 characterize short N⋯H/H⋯N contacts, and near atoms H19 and C24 indicate short H⋯C/C⋯H contacts. The relative distributions from the different inter­atomic contacts to the Hirshfeld surfaces are summarized in Table 3 ▸. The largest contributions are contacts in which H atoms are involved. The largest differences between both orientations are observed for H⋯S/S⋯H (9.5%), H⋯H (5.7%), S⋯S (3.3%) and C⋯S/S⋯C (3.1%) contacts, and are caused by the presence of the C26—H26⋯S15^ii^ hydrogen bond in orientation B.

## Database survey   

A search of the Cambridge Structral Database (CSD, Version 3.38, last update May 2017; Groom *et al.*, 2016 ▸) for 3-(benzo­thia­zol-2-yl)thio­phene derivatives gives two hits: 2-anilino-4-(1,3-benzo­thia­zol-2-yl)-5-(4-chloro­benzo­yl)thio­phene-3-carbo­nitrile (refcode LEGHOW; Fun *et al.*, 2012 ▸) and 3-(1,3-benzo­thia­zol-2-yl)-*N*-(quinolin-8-yl)thio­phene-2-carboxamide (refcode UVUGOJ; Cheng *et al.*, 2016 ▸). The substitution of the thio­phene ring in these two compounds has an influence on the angle between the best planes through the thio­phene and benzo­thia­zole rings. In the monosubstituted derivative UVUGOJ, an intra­molecular N—H⋯S hydrogen bond lowers the angle to 5.95°. For the tris­ubstituted derivative LEGHOW, the angle increases to 46.77°.

## Synthesis and crystallization   

**Figure 6:**
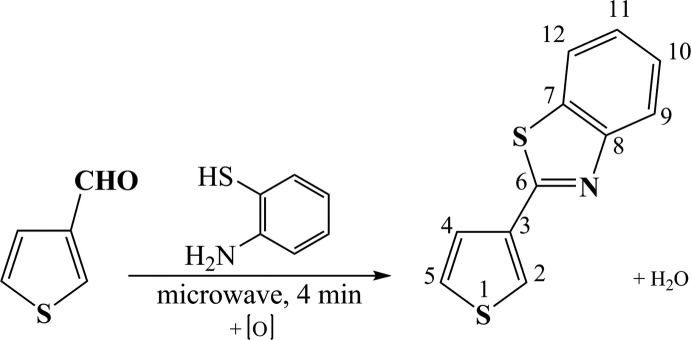
Reaction scheme for the title compound.

The reaction scheme to synthesize the title compound is given in Fig. 6 ▸. The reaction mechanism is similar to that described by Mukhopadhyay & Datta (2007 ▸) for the synthesis of 2-aryl­benzo­thia­zoles.

A reaction mixture of thio­phene-3-carbaldehyde (2 mmol) and *o*-amino­thio­phenol (2 mmol) was heated for 4 min in a domestic microwave (Sanyo EM-S1065, 800 W) at medium power level (400 W). The progress of the reaction was monitored with thin-layer chromatography (TLC) every minute. The mixture was cooled to room temperature and then dissolved in an *n*-hexa­ne–ethyl acetate mixture (5:1 *v*/*v*) to obtain a solid product, which was further crystallized in the same solvent to give 0.38 g (yield 87%) of the title product as pale-yellow crystals (m.p. 386 K). IR (Nicolet Impact 410 FT–IR, KBr, cm^−1^): 3067 (ν_CH_), 1581 (ν_C=C_), 1634 (ν_C=N_). ^1^H NMR [Bruker XL-500, 500 MHz, *d*_6_-DMSO, δ (ppm), *J* (Hz)]: 8.36 (*dd*, 1H, ^4^ *J* = 1.0, ^5^ *J* = 2.5, H^2^), 7.72 (*dd*, 1H, ^2^ *J* = 1.0, ^5^ *J* = 5.0, H^4^), 7.77 (*dd*, 1H, ^2^ *J* = 2.5, ^4^ *J* = 5.0, H^5^), 8.02 (*dd*, 1H, ^11^ *J* = 1.0, ^10^ *J* = 8.0, H^9^), 7.52 (*td*, 1H, ^12^ *J* = 1.0, ^11^ *J* = 7.5, ^9^ *J* = 8.0, H^10^), 7.44 (*td*, 1H, ^9^ *J* = 1.0, ^10^ *J* = 7.5, ^12^ *J* = 8.0, H^11^), 8.11 (*dd*, 1H, ^10^ *J* = 1.0, ^11^ *J* = 8.0, H^12^). ^13^C NMR [Bruker XL-500, 125 MHz, *d*_6_-DMSO, δ (ppm)]: 127.54 (C^2^), 135.17 (C^3^),126.17 (C^4^), 128.38 (C^5^), 162.17 (C^6^), 134.17 (C^7^), 153.30 (C^8^), 122.57 (C^9^), 126.53 (C^10^), 125.30 (C^11^), 122.22 (C^12^). Calculation for C_11_H_7_NS_2_: *M* = 217 a.u.

## Refinement   

Crystal data, data collection and structure refinement details are summarized in Table 4 ▸. The mol­ecule is disordered over two positions (*A* and *B*) by a rotation of approximately 180°. The final occupancy factors are 0.4884 (10) for mol­ecule *A* and 0.5116 (10) for mol­ecule *B*. Enhanced rigid-body restraints (RIGU) were applied for all atoms. The H atoms were placed in idealized positions and refined in riding mode, with *U*_iso_(H) values assigned as 1.2*U*_eq_ of the parent atoms, with a C—H distance of 0.95 Å. In the final cycles of refinement, 17 outliers were omitted.

## Supplementary Material

Crystal structure: contains datablock(s) I. DOI: 10.1107/S2056989017014530/tx2001sup1.cif

Structure factors: contains datablock(s) I. DOI: 10.1107/S2056989017014530/tx2001Isup2.hkl

CCDC reference: 1578811

Additional supporting information:  crystallographic information; 3D view; checkCIF report

## Figures and Tables

**Table 1:** Selected π–π inter­actions *Cg*1 is the centroid of the S15/C16–C19 plane, *Cg*2 that of the C20/S21/C22/C27/N28 plane, *Cg*3 that of the C22–C27 plane, *Cg*4 that of the S1/C2–C5 plane, *Cg*5 that of the C6/S7/C8/C13/N14 plane and *Cg*6 that of the C8–C13 plane.

*CgI*	*CgJ*	*Cg*–*Cg* (Å)	α (°)	*CgI*_Perp (Å)	*CgJ*_Perp (Å)
*Cg*1	*Cg*2^i^	3.888 (3)	12.0 (2)	3.761 (2)	−3.7335 (17)
*Cg*1	*Cg*3^i^	3.962 (3)	13.0 (2)	3.774 (2)	−3.614 (2)
*Cg*2	*Cg*1^ii^	3.888 (3)	12.0 (2)	−3.7335 (17)	3.761 (2)
*Cg*2	*Cg*6^ii^	3.973 (3)	9.4 (2)	−3.6796 (17)	3.708 (2)
*Cg*3	*Cg*1^ii^	3.962 (3)	13.0 (2)	−3.614 (2)	3.774 (2)
*Cg*3	*Cg*6^ii^	3.799 (3)	10.4 (2)	−3.631 (2)	3.720 (2)
*Cg*4	*Cg*5^ii^	3.859 (3)	9.6 (2)	−3.5981 (19)	3.7215 (17)
*Cg*4	*Cg*6^ii^	3.882 (3)	10.4 (2)	−3.5850 (19)	3.674 (2)
*Cg*5	*Cg*4^i^	3.859 (3)	9.6 (2)	3.7215 (17)	−3.5981 (19)
*Cg*6	*Cg*2^i^	3.972 (3)	9.4 (2)	3.708 (2)	−3.6796 (17)
*Cg*6	*Cg*3^i^	3.798 (3)	10.4 (2)	3.719 (2)	−3.631 (2)
*Cg*6	*Cg*4^i^	3.882 (3)	10.4 (2)	3.673 (2)	−3.5851 (19)

Notes: *CgI*(*J*) = plane number *I*(*J*); *Cg*–*Cg* = distance between ring centroids; *CgI*_Perp = perpendicular distance of *CgI* on ring *J*; *CgJ*_Perp = perpendicular distance of *CgJ* on ring *I*. Symmetry codes: (i) *x* + 1, *y*, *z*; (ii) *x* − 1, *y*, *z*.

**Table 2:** Hydrogen-bond geometry (Å, °) *Cg*1 is the centroid of the S15/C16–C19 plane, *Cg*3 that of the C22–C27 plane, *Cg*4 that of the S1/C2–C5 plane and *Cg*6 that of the C8–C13 plane.

*D*—H⋯*A*	*D*—H	H⋯*A*	*D*⋯*A*	*D*—H⋯*A*
C9—H9⋯N14^i^	0.95	2.54	3.355 (6)	144
C26—H26⋯S15^ii^	0.95	2.87	3.522 (5)	126
C5—H5⋯*Cg*1^iii^	0.95	2.86	3.496 (5)	125
C5—H5⋯*Cg*6^iii^	0.95	2.93	3.532 (5)	123
C11—H11⋯*Cg*3^iv^	0.95	2.90	3.670 (6)	139
C11—H11⋯*Cg*4^iv^	0.95	2.90	3.705 (6)	143
C19—H19⋯*Cg*3^iv^	0.95	2.74	3.418 (6)	129
C19—H19⋯*Cg*4^iv^	0.95	2.73	3.447 (6)	133

Symmetry codes: (i) 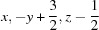; (ii) 
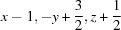
; (iii) 
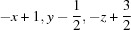
; (iv) 
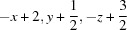
.

**Table 3:** Percentage contributions of inter­atomic contacts to the Hirshfeld surfaces

Contact	Orientation A	Orientation B
H⋯H	35.8	30.1
S⋯H/H⋯S	15.9	25.4
C⋯H/H⋯C	20.2	21.8
N⋯H/H⋯N	6.4	7.7
C⋯C	8.0	8.9
C⋯S/S⋯C	6.1	3.0
S⋯S	4.2	0.9
S⋯N/N⋯S	2.3	1.1
C⋯N/N⋯C	1.0	1.1

**Table 4:** Experimental details

Crystal data	
Chemical formula	C_11_H_7_NS_2_
*M*_r_	217.30
Crystal system, space group	Monoclinic, *P*2_1_/*c*
Temperature (K)	100
*a*, *b*, *c* (Å)	6.1368 (4), 13.9799 (9), 11.4609 (7)
β (°)	100.193 (2)
*V* (Å^3^)	967.73 (11)
*Z*	4
Radiation type	Mo *K*α
μ (mm^−1^)	0.50
Crystal size (mm)	0.44 × 0.36 × 0.31

Data collection	
Diffractometer	Bruker APEXII CCD
Absorption correction	Multi-scan (*SADABS*; Bruker, 2014 ▸)
*T*_min_, *T*_max_	0.703, 0.747
No. of measured, independent and observed [*I* > 2σ(*I*)] reflections	19256, 2385, 2255
*R*_int_	0.034
(sin θ/λ)_max_ (Å^−1^)	0.667

Refinement	
*R*[*F* ^2^ > 2σ(*F* ^2^)], *wR*(*F* ^2^), *S*	0.076, 0.172, 1.22
No. of reflections	2385
No. of parameters	254
No. of restraints	228
H-atom treatment	H-atom parameters constrained
Δρ_max_, Δρ_min_ (e Å^−3^)	0.61, −0.52

Computer programs: *APEX2* (Bruker, 2014 ▸), *SAINT* (Bruker, 2013 ▸), *SHELXT2016* (Sheldrick, 2015*a* ▸), *SHELXL2014* (Sheldrick, 2015*b* ▸) and *OLEX2* (Dolomanov *et al.*, 2009 ▸).

## supplementary crystallographic information

### Crystal data

**Table:**

C_11_H_7_NS_2_	*F*(000) = 448
*M**_r_* = 217.30	*D*_x_ = 1.491 Mg m^−^^3^
Monoclinic, *P*2_1_/*c*	Mo *K*α radiation, λ = 0.71073 Å
*a* = 6.1368 (4) Å	Cell parameters from 9904 reflections
*b* = 13.9799 (9) Å	θ = 2.9–32.6°
*c* = 11.4609 (7) Å	µ = 0.50 mm^−^^1^
β = 100.193 (2)°	*T* = 100 K
*V* = 967.73 (11) Å^3^	Block, colorless
*Z* = 4	0.44 × 0.36 × 0.31 mm

### Data collection

**Table:**

Bruker APEXII CCD diffractometer	2255 reflections with *I* > 2σ(*I*)
φ and ω scans	*R*_int_ = 0.034
Absorption correction: multi-scan (SADABS; Bruker, 2014)	θ_max_ = 28.3°, θ_min_ = 2.9°
*T*_min_ = 0.703, *T*_max_ = 0.747	*h* = −8→8
19256 measured reflections	*k* = −18→18
2385 independent reflections	*l* = −15→15

### Refinement

**Table:**

Refinement on *F*^2^	228 restraints
Least-squares matrix: full	Hydrogen site location: inferred from neighbouring sites
*R*[*F*^2^ > 2σ(*F*^2^)] = 0.076	H-atom parameters constrained
*wR*(*F*^2^) = 0.172	*w* = 1/[σ^2^(*F*_o_^2^) + (0.0293*P*)^2^ + 2.8579*P*] where *P* = (*F*_o_^2^ + 2*F*_c_^2^)/3
*S* = 1.22	(Δ/σ)_max_ = 0.001
2385 reflections	Δρ_max_ = 0.61 e Å^−^^3^
254 parameters	Δρ_min_ = −0.52 e Å^−^^3^

### Special details

**Table:**

Geometry. All esds (except the esd in the dihedral angle between two l.s. planes) are estimated using the full covariance matrix. The cell esds are taken into account individually in the estimation of esds in distances, angles and torsion angles; correlations between esds in cell parameters are only used when they are defined by crystal symmetry. An approximate (isotropic) treatment of cell esds is used for estimating esds involving l.s. planes.

### Fractional atomic coordinates and isotropic or equivalent isotropic displacement parameters (Å^2^)

**Table:**

	*x*	*y*	*z*	*U*_iso_*/*U*_eq_	Occ. (<1)
S1	0.1359 (2)	0.50570 (11)	0.64696 (12)	0.0370 (3)	0.4884 (10)
C2	0.3260 (9)	0.5734 (4)	0.5993 (4)	0.0304 (8)	0.4884 (10)
H2	0.332074	0.581845	0.517697	0.036*	0.4884 (10)
C3	0.4732 (7)	0.6159 (3)	0.6887 (4)	0.0208 (6)	0.4884 (10)
C4	0.4069 (8)	0.5922 (3)	0.8006 (4)	0.0242 (7)	0.4884 (10)
H4	0.478997	0.617848	0.874094	0.029*	0.4884 (10)
C5	0.2230 (7)	0.5268 (3)	0.7915 (4)	0.0218 (7)	0.4884 (10)
H5	0.162696	0.500659	0.855346	0.026*	0.4884 (10)
C6	0.6552 (7)	0.6774 (3)	0.6747 (4)	0.0202 (6)	0.4884 (10)
S7	0.68433 (19)	0.71644 (9)	0.53194 (10)	0.0230 (2)	0.4884 (10)
C8	0.9176 (7)	0.7805 (3)	0.5945 (3)	0.0188 (6)	0.4884 (10)
C9	1.0540 (8)	0.8389 (4)	0.5407 (4)	0.0256 (7)	0.4884 (10)
H9	1.026389	0.849650	0.457569	0.031*	0.4884 (10)
C10	1.2293 (8)	0.8799 (4)	0.6130 (4)	0.0294 (8)	0.4884 (10)
H10	1.325861	0.919477	0.577890	0.035*	0.4884 (10)
C11	1.2752 (8)	0.8670 (4)	0.7362 (4)	0.0267 (8)	0.4884 (10)
H11	1.398885	0.897336	0.783337	0.032*	0.4884 (10)
C12	1.1363 (8)	0.8092 (4)	0.7873 (4)	0.0246 (7)	0.4884 (10)
H12	1.163974	0.799310	0.870616	0.029*	0.4884 (10)
C13	0.9581 (7)	0.7659 (3)	0.7183 (4)	0.0196 (6)	0.4884 (10)
N14	0.8042 (6)	0.7076 (3)	0.7618 (3)	0.0213 (6)	0.4884 (10)
S15	1.2597 (2)	0.90620 (10)	0.59226 (10)	0.0325 (3)	0.5116 (10)
C16	1.0318 (8)	0.8344 (4)	0.5615 (4)	0.0260 (8)	0.5116 (10)
H16	0.946788	0.825702	0.484562	0.031*	0.5116 (10)
C17	0.9864 (6)	0.7905 (3)	0.6624 (3)	0.0204 (6)	0.5116 (10)
C18	1.1510 (8)	0.8185 (3)	0.7658 (4)	0.0247 (7)	0.5116 (10)
H18	1.149066	0.795829	0.843803	0.030*	0.5116 (10)
C19	1.3063 (8)	0.8798 (4)	0.7391 (4)	0.0255 (8)	0.5116 (10)
H19	1.425444	0.904712	0.795189	0.031*	0.5116 (10)
C20	0.8024 (7)	0.7272 (3)	0.6680 (4)	0.0216 (6)	0.5116 (10)
S21	0.6485 (2)	0.68370 (9)	0.53382 (10)	0.0282 (3)	0.5116 (10)
C22	0.4817 (7)	0.6220 (3)	0.6160 (4)	0.0255 (6)	0.5116 (10)
C23	0.3070 (8)	0.5607 (4)	0.5775 (5)	0.0319 (8)	0.5116 (10)
H23	0.260774	0.544164	0.496484	0.038*	0.5116 (10)
C24	0.2039 (10)	0.5250 (4)	0.6684 (5)	0.0466 (10)	0.5116 (10)
H24	0.078484	0.485473	0.643384	0.056*	0.5116 (10)
C25	0.2579 (7)	0.5390 (3)	0.7860 (5)	0.0269 (7)	0.5116 (10)
H25	0.175882	0.512781	0.841294	0.032*	0.5116 (10)
C26	0.4498 (7)	0.5970 (4)	0.8198 (4)	0.0276 (7)	0.5116 (10)
H26	0.506396	0.606303	0.901659	0.033*	0.5116 (10)
C27	0.5547 (7)	0.6395 (3)	0.7374 (4)	0.0227 (6)	0.5116 (10)
N28	0.7402 (6)	0.6993 (3)	0.7626 (3)	0.0235 (6)	0.5116 (10)

### Atomic displacement parameters (Å^2^)

**Table:**

	*U*^11^	*U*^22^	*U*^33^	*U*^12^	*U*^13^	*U*^23^
S1	0.0362 (6)	0.0388 (7)	0.0338 (6)	−0.0082 (5)	0.0007 (5)	0.0022 (5)
C2	0.0364 (15)	0.0304 (19)	0.0229 (11)	−0.0075 (11)	0.0016 (10)	−0.0026 (12)
C3	0.0204 (10)	0.0211 (14)	0.0209 (10)	0.0033 (8)	0.0036 (8)	−0.0010 (10)
C4	0.0273 (13)	0.0233 (16)	0.0227 (10)	0.0023 (10)	0.0062 (9)	−0.0004 (11)
C5	0.0224 (13)	0.0164 (15)	0.0276 (11)	0.0063 (10)	0.0069 (11)	−0.0006 (12)
C6	0.0221 (10)	0.0205 (14)	0.0180 (10)	0.0019 (8)	0.0038 (8)	−0.0019 (10)
S7	0.0251 (5)	0.0246 (5)	0.0180 (4)	−0.0042 (4)	0.0004 (4)	0.0005 (4)
C8	0.0206 (11)	0.0185 (14)	0.0172 (9)	0.0014 (8)	0.0033 (8)	−0.0023 (9)
C9	0.0310 (13)	0.0225 (16)	0.0257 (12)	−0.0041 (10)	0.0115 (9)	−0.0031 (11)
C10	0.0341 (15)	0.0267 (19)	0.0296 (10)	−0.0065 (12)	0.0116 (10)	−0.0033 (12)
C11	0.0245 (14)	0.0281 (18)	0.0286 (10)	−0.0022 (11)	0.0076 (10)	−0.0038 (12)
C12	0.0242 (12)	0.0293 (16)	0.0205 (12)	−0.0030 (9)	0.0048 (9)	−0.0046 (11)
C13	0.0221 (10)	0.0188 (14)	0.0178 (9)	0.0022 (8)	0.0029 (8)	−0.0004 (9)
N14	0.0224 (10)	0.0225 (14)	0.0185 (10)	0.0005 (9)	0.0020 (8)	0.0000 (10)
S15	0.0324 (5)	0.0418 (6)	0.0241 (4)	−0.0105 (5)	0.0072 (4)	−0.0022 (5)
C16	0.0259 (14)	0.0317 (17)	0.0198 (10)	−0.0032 (11)	0.0026 (10)	−0.0032 (11)
C17	0.0209 (10)	0.0203 (13)	0.0200 (10)	0.0019 (8)	0.0037 (8)	−0.0057 (9)
C18	0.0268 (12)	0.0271 (16)	0.0198 (11)	−0.0038 (10)	0.0030 (9)	−0.0034 (11)
C19	0.0267 (13)	0.0279 (16)	0.0215 (11)	−0.0044 (10)	0.0033 (11)	−0.0019 (12)
C20	0.0200 (10)	0.0206 (13)	0.0233 (10)	0.0012 (8)	0.0009 (8)	−0.0030 (9)
S21	0.0306 (5)	0.0311 (6)	0.0203 (4)	−0.0078 (4)	−0.0028 (4)	0.0016 (4)
C22	0.0258 (12)	0.0208 (14)	0.0279 (10)	−0.0029 (9)	−0.0006 (9)	−0.0009 (10)
C23	0.0302 (14)	0.0231 (16)	0.0374 (13)	−0.0065 (10)	−0.0075 (10)	0.0021 (12)
C24	0.0482 (19)	0.044 (2)	0.0448 (11)	−0.0254 (15)	0.0000 (10)	−0.0028 (12)
C25	0.0213 (13)	0.0174 (16)	0.0412 (11)	0.0018 (10)	0.0029 (11)	0.0000 (13)
C26	0.0242 (12)	0.0260 (16)	0.0327 (12)	−0.0040 (10)	0.0054 (9)	−0.0013 (11)
C27	0.0204 (11)	0.0207 (14)	0.0261 (9)	0.0013 (8)	0.0017 (8)	−0.0018 (9)
N28	0.0234 (11)	0.0225 (13)	0.0242 (9)	−0.0012 (9)	0.0032 (8)	−0.0032 (9)

### Geometric parameters (Å, º)

**Table:**

S1a—C2	1.667 (6)	S15b—C16	1.707 (5)
C2a—H2	0.9500	C16b—H16	0.9500
C2a—C3	1.375 (6)	C16b—C17	1.380 (6)
C3a—C4	1.449 (6)	C17b—C18	1.468 (6)
C4a—H4	0.9500	C18b—H18	0.9500
S1a—C5	1.674 (5)	S15b—C19	1.697 (5)
C4a—C5	1.442 (7)	C18b—C19	1.357 (7)
C5a—H5	0.9500	C19b—H19	0.9500
C3a—C6	1.442 (6)	C17b—C20	1.444 (6)
C6a—S7	1.763 (4)	C20b—S21	1.764 (4)
S7a—C8	1.733 (4)	S21b—C22	1.738 (5)
C8a—C9	1.389 (6)	C22b—C23	1.383 (6)
C9a—H9	0.9500	C23b—H23	0.9500
C9a—C10	1.363 (7)	C23b—C24	1.403 (8)
C10a—H10	0.9500	C24b—H24	0.9500
C10a—C11	1.402 (7)	C24b—C25	1.344 (8)
C11a—H11	0.9500	C25b—H25	0.9500
C11a—C12	1.378 (7)	C25b—C26	1.426 (6)
C12a—H12	0.9500	C26b—H26	0.9500
C12a—C13	1.372 (6)	C26b—C27	1.369 (7)
C8a—C13	1.411 (5)	C22b—C27	1.406 (6)
C6a—N14	1.299 (5)	C20b—N28	1.273 (6)
C13a—N14	1.404 (6)	C27b—N28	1.401 (5)
			
C3a—C2a—S1	114.0 (4)	C18b—C19b—S15	111.1 (3)
C4a—C5a—S1	107.0 (3)	C17b—C16b—S15	111.6 (3)
C3a—C2a—H2	123.0	C19b—S15b—C16	93.7 (2)
S1a—C2a—H2	123.0	C17b—C16b—H16	124.2
N14a—C6a—C3	124.2 (4)	S15b—C16b—H16	124.2
C5a—C4a—C3	114.8 (4)	N28b—C20b—C17	125.4 (4)
C2a—C3a—C4	108.1 (4)	C19b—C18b—C17	113.4 (4)
C6a—C3a—C4	125.4 (4)	C20b—C17b—C18	124.0 (4)
C5a—C4a—H4	122.6	C16b—C17b—C18	110.2 (4)
C3a—C4a—H4	122.6	C19b—C18b—H18	123.3
C2a—S1a—C5	96.0 (2)	C17b—C18b—H18	123.3
C4a—C5a—H5	126.5	S15b—C19b—H19	124.5
S1a—C5a—H5	126.5	C18b—C19b—H19	124.5
C2a—C3a—C6	126.4 (4)	C16b—C17b—C20	125.8 (4)
C8a—S7a—C6	89.31 (19)	C22b—S21b—C20	88.5 (2)
C3a—C6a—S7	119.8 (3)	C17b—C20b—S21	118.4 (3)
N14a—C6a—S7	116.1 (3)	N28b—C20b—S21	116.2 (3)
C9a—C8a—S7	129.7 (3)	C23b—C22b—S21	129.2 (4)
C13a—C8a—S7	109.1 (3)	C27b—C22b—S21	109.6 (3)
C10a—C9a—C8	116.8 (4)	C26b—C27b—C22	120.0 (4)
N14a—C13a—C8	115.5 (4)	N28b—C27b—C22	114.4 (4)
C12a—C13a—C8	119.7 (4)	C25b—C24b—C23	129.0 (5)
C8a—C9a—H9	121.6	C24b—C23b—H23	122.9
C10a—C9a—H9	121.6	C22b—C23b—H23	122.9
C12a—C11a—C10	118.3 (4)	C22b—C23b—C24	114.1 (5)
C11a—C10a—H10	118.2	C23b—C24b—H24	115.5
C9a—C10a—H10	118.2	C25b—C24b—H24	115.5
C9a—C10a—C11	123.6 (5)	C27b—C26b—C25	121.7 (4)
C13a—C12a—C11	120.3 (4)	C26b—C25b—H25	123.2
C12a—C11a—H11	120.8	C24b—C25b—H25	123.2
C10a—C11a—H11	120.8	C24b—C25b—C26	113.6 (5)
C11a—C12a—H12	119.9	C25b—C26b—H26	119.1
C13a—C12a—H12	119.9	C27b—C26b—H26	119.1
C9a—C8a—C13	121.2 (4)	C20b—N28b—C27	111.3 (4)
C6a—N14a—C13	110.0 (4)	C23b—C22b—C27	121.1 (4)
C12a—C13a—N14	124.7 (4)	C26b—C27b—N28	125.6 (4)
			
C5a—S1a—C2a—C3a	1.2 (4)	C19b—S15b—C16b—C17b	0.8 (4)
S1a—C2a—C3a—C6a	179.5 (4)	S15b—C16b—C17b—C20b	178.4 (3)
S1a—C2a—C3a—C4a	−3.4 (5)	S15b—C16b—C17b—C18b	−0.7 (5)
C2a—C3a—C4a—C5a	4.7 (6)	C16b—C17b—C18b—C19b	0.1 (6)
C6a—C3a—C4a—C5a	−178.3 (4)	C20b—C17b—C18b—C19b	−178.9 (4)
C3a—C4a—C5a—S1a	−3.8 (5)	C17b—C18b—C19b—S15b	0.5 (5)
C2a—S1a—C5a—C4a	1.5 (4)	C16b—S15b—C19b—C18b	−0.7 (4)
C2a—C3a—C6a—N14a	−172.3 (5)	C16b—C17b—C20b—N28b	−167.9 (5)
C4a—C3a—C6a—N14a	11.2 (7)	C18b—C17b—C20b—N28b	11.0 (7)
C2a—C3a—C6a—S7a	8.0 (6)	C16b—C17b—C20b—S21b	12.2 (6)
C4a—C3a—C6a—S7a	−168.5 (4)	C18b—C17b—C20b—S21b	−168.9 (3)
N14a—C6a—S7a—C8a	−0.5 (4)	N28b—C20b—S21b—C22b	0.4 (4)
C3a—C6a—S7a—C8a	179.2 (4)	C17b—C20b—S21b—C22b	−179.7 (3)
C6a—S7a—C8a—C9a	−178.7 (4)	C20b—S21b—C22b—C23b	−177.2 (5)
C6a—S7a—C8a—C13a	1.2 (3)	C20b—S21b—C22b—C27b	0.3 (3)
C13a—C8a—C9a—C10a	0.8 (7)	C27b—C22b—C23b—C24b	4.4 (7)
S7a—C8a—C9a—C10a	−179.3 (4)	S21b—C22b—C23b—C24b	−178.3 (4)
C8a—C9a—C10a—C11a	−0.8 (8)	C22b—C23b—C24b—C25b	−3.2 (9)
C9a—C10a—C11a—C12a	0.4 (8)	C23b—C24b—C25b—C26b	−1.3 (9)
C10a—C11a—C12a—C13a	0.0 (8)	C24b—C25b—C26b—C27b	4.9 (7)
C11a—C12a—C13a—N14a	−178.5 (4)	C25b—C26b—C27b—N28b	178.3 (4)
C11a—C12a—C13a—C8a	0.1 (7)	C25b—C26b—C27b—C22b	−3.8 (7)
C9a—C8a—C13a—C12a	−0.5 (7)	C23b—C22b—C27b—C26b	−1.2 (7)
S7a—C8a—C13a—C12a	179.6 (4)	S21b—C22b—C27b—C26b	−179.0 (4)
C9a—C8a—C13a—N14a	178.2 (4)	C23b—C22b—C27b—N28b	176.9 (4)
S7a—C8a—C13a—N14a	−1.7 (5)	S21b—C22b—C27b—N28b	−0.9 (5)
C3a—C6a—N14a—C13a	179.9 (4)	C17b—C20b—N28b—C27b	179.1 (4)
S7a—C6a—N14a—C13a	−0.3 (5)	S21b—C20b—N28b—C27b	−1.0 (5)
C12a—C13a—N14a—C6a	180.0 (4)	C26b—C27b—N28b—C20b	179.2 (4)
C8a—C13a—N14a—C6a	1.3 (5)	C22b—C27b—N28b—C20b	1.2 (5)

### Hydrogen-bond geometry (Å, º)

*Cg*1 is the centroid of the S15/C16–C19 plane, *Cg*3 that of the C22–C27 plane, *Cg*4 that of the S1/C2–C5 plane and *Cg*6 that of the C8–C13 plane.

**Table:**

*D*—H···*A*	*D*—H	H···*A*	*D*···*A*	*D*—H···*A*
C9—H9···N14^i^	0.95	2.54	3.355 (6)	144
C26—H26···S15^ii^	0.95	2.87	3.522 (5)	126
C5—H5···*Cg*1^iii^	0.95	2.86	3.496 (5)	125
C5—H5···*Cg*6^iii^	0.95	2.93	3.532 (5)	123
C11—H11···*Cg*3^iv^	0.95	2.90	3.670 (6)	139
C11—H11···*Cg*4^iv^	0.95	2.90	3.705 (6)	143
C19—H19···*Cg*3^iv^	0.95	2.74	3.418 (6)	129
C19—H19···*Cg*4^iv^	0.95	2.73	3.447 (6)	133

Symmetry codes: (i) *x*, −*y*+3/2, *z*−1/2; (ii) *x*−1, −*y*+3/2, *z*+1/2; (iii) −*x*+1, *y*−1/2, −*z*+3/2; (iv) −*x*+2, *y*+1/2, −*z*+3/2.
